# The efficacy of sensory neural entrainment on acute and chronic pain: A systematic review and meta-analysis

**DOI:** 10.1177/20494637221139472

**Published:** 2022-11-10

**Authors:** Rhys Maddison, Hamde Nazar, Ilona Obara, Quoc C Vuong

**Affiliations:** 1 School of Pharmacy, 5994 Newcastle University, Newcastle Upon Tyne, UK; 2 Population and Health Sciences Institute, 5994 Newcastle University, Newcastle Upon Tyne, UK; 3 Translational and Clinical Research Institute, 5994 Newcastle University, Newcastle Upon Tyne, UK; 4 Biosciences Institute, 5994 Newcastle University, Newcastle Upon Tyne, UK; 5 School of Psychology, 5994 Newcastle University, Newcastle Upon Tyne, UK

**Keywords:** acute pain, chronic pain, neural oscillation, sensory entrainment, binaural beat

## Abstract

**Background:**

Changes to the power of neural oscillations in cortical and sub-cortical structures can change pain perception. Rhythmic sensory stimulation is a non-invasive method that can increase power in specific frequencies of neural oscillations. If the stimulation frequency targets those frequencies related to pain perception, such as alpha or theta frequencies, there can be a reduction in perceived pain intensity. Thus, sensory neural entrainment may provide an alternative to pharmacological intervention for acute and chronic pain. This review aimed to identify and critically appraise the evidence on the effectiveness of sensory entrainment methods for pain perception.

**Methods:**

We undertook a systematic search across Medline, Embase, PsycInfo, Web of Science and Scopus in November 2020 to identify studies investigating the efficacy of sensory entrainment on adults. We assessed studies for their quality using the PRISMA checklist. A random-effects model was used in a meta-analysis to measure the effect of entrainment on pain perception.

**Results:**

Our systematic review yielded nine studies fitting the search criteria. Studies investigated the effect of visual and auditory entrainment on pain intensity rating, electrophysiological markers of pain and amount of analgesia needed during surgery. The meta-analysis suggests that alpha (8–13 Hz) sensory entrainment is effective for acute pain perception, whereas theta (4–7 Hz) entrainment is effective for chronic pain.

**Conclusions:**

Although there is heterogeneity in the current evidence, our review highlights the potential use of sensory entrainment to affect acute and chronic pain. Further research is required regarding the timing, duration and frequency of the stimulation to determine the best application for maximum efficacy.

## Introduction

Pharmacological interventions are often used to reduce perceived pain intensity but may have adverse side-effects or lack long-term analgesic efficacy.^1,2^ Interventions that avoid these issues can provide safer methods to reduce pain intensity. There is evidence that neural oscillations of cortical and sub-cortical structures at specific frequencies contribute to pain perception.^3^ These oscillations reflect rhythmic fluctuations of neural activity in the brain. Experimental studies using electroencephalography (EEG) have shown pain-related changes to neural oscillations in theta (4–7 Hz), alpha (8–13 Hz), beta (14–29 Hz) and gamma (30–100 Hz) frequency bands. Power in these bands change during painful stimulation, such as a reduction in alpha power or an increase in theta and gamma power to noxious laser, heat or cold stimulation.^4–11^ In addition, chronic-pain patients show abnormal changes in neural oscillations.^12–14^ The role of neural oscillations in pain perception suggests that sensory neural entrainment may help pain management.^15^

Sensory entrainment uses rhythmic external stimulation to synchronise neural oscillations to the stimulation frequency and increase power at that frequency.^16–18^ We hypothesise that neural entrainment induced by sensory stimulation changes individuals’ pain state by affecting power in oscillation frequencies associated with pain perception.^3,19^ Visual entrainment uses visual patterns flashing at a desired frequency, for example, LEDs flashing at 10 Hz. This stimulation has the largest effect on alpha power in visual cortex but can also engage pain-related areas including parietal, cingulate and insular cortices.^20^ Auditory entrainment predominantly uses binaural beats to present auditory stimulation at specific frequencies. They are generated by presenting pure tones of different frequencies to the left and right ear, resulting in an illusory beat corresponding to the difference between the two frequencies. For example, a 310 Hz pure tone presented to the left ear and a 320 Hz pure tone presented to the right ear would result in the perception of a 10 Hz beat. Binaural-beat perception is thought to take place in the brain stem and superior olivary nucleus of each cerebral hemisphere moving on to the reticular formations,^21^ and to promote hemispheric synchronisation. The existing research on tactile entrainment is limited, only showing effects of slow brushing to reduce pain.^22^

### Aim

The use of sensory entrainment methods may provide non-pharmacological interventions for pain management.^16,23–24^ One previous meta-analysis focused on binaural beats on cognitive and psychological states, including pain.^26^ However, there are currently no systematic reviews or meta-analyses synthesising and critiquing the evidence about the effectiveness of sensory entrainment methods for pain perception. Our study addresses this gap by critically appraising the current evidence on the efficacy of sensory entrainment in reducing adults’ subjective pain intensity.

## Method

This study complies with the Preferred Reporting Items for Systematic Reviews and Meta-Analyses (PRISMA) criteria.^27^ The search strategy was refined in consultation with an expert librarian. The review and protocol was not registered with a recognised database.

### Search strategy

In November 2020, five electronic databases were systematically searched with no date limits applied: Medline, Embase, PsycInfo, Web of Science and Scopus. Keywords and their synonyms were used with ‘AND’ and ‘OR’ to narrow or broaden the search depending on the search strategy for each database. The electronic searches were followed by hand citation searches and reference list review to identify additional studies. The search was limited to the English language and only included published studies.

### Study eligibility

The population, intervention, control, outcomes and studies (PICOS) framework^28^ was used to specify the inclusion and exclusion criteria and inform the key search terms used. The PICOS components are presented in Table 1.

**Table 1.** The PICOS components of the research question and respective search terms used.

PICOS component	Description	Key search terms
Population	Human adults experiencing and not experiencing pain	—
Intervention	Sensory entrainment including auditory, visual and tactile	‘Sensory entrainment’ OR ‘auditory entrainment’ OR ‘sound entrainment’ OR ‘acoustic entrainment’ OR ‘audiovisual entrainment’ OR ‘music entrainment’ OR ‘visual entrainment’ OR ‘photic entrainment’ OR ‘light entrainment’ OR ‘tactile entrainment’ OR ‘touch entrainment’ OR ‘vibration entrainment’ OR ‘monaural beats’ OR ‘binaural beats’ OR ‘isochronic beats’ OR ‘isochronic tones’ OR ‘binaural tones’ OR ‘monaural tones’ OR ‘binaural sounds’ OR ‘monaural sounds’ OR ‘isochronic sounds’ OR ‘flashing light’ OR ‘flashing screen’ OR ‘LED glasses’ OR ‘flashes of light’ OR ‘light flashes’ OR ‘pitch panning’ OR ‘slow brushing’ OR ‘fast brushing’ OR ‘brainwave entrainment’ OR ‘oscillatory entrainment’ OR ‘neural entrainment’ OR ‘light pulses’ OR ‘gamma entrainment’ OR ‘alpha entrainment’ OR ‘beta entrainment’ or ‘theta entrainment’ OR ‘delta entrainment’ OR ‘hemi-sync’ OR ‘hemisync’ OR ‘hemispheric synchronised’ OR ‘auditory beats’ OR ‘vibration therapy’ OR ‘rhythmic stimulation’ OR ‘rhythmic sensory stimulation’
Control	Sham stimulation, control stimulation, no stimulation	—
Outcomes	Pain perception and intensity	‘Pain perception’ OR ‘pain threshold’ OR ‘pain measurement’ OR ‘pain management’ OR ‘pain assessment’ OR ‘pain severity’ OR ‘analgesia’ OR ‘analgesic effect’ OR ‘analgesic activity’ OR ‘nociception’ OR ‘antinociceptive’ OR ‘visual analogue scale’ OR ‘numerical rating scale’
Studies	Experimental and observational studies	—

### Study selection

One author [RM] undertook the initial searching and screening of abstracts. All other authors were consulted when there was doubt of eligibility. The full texts were read and screened by at least two authors [RM and HN/IO/QV]. Data abstraction and quality assessment were undertaken independently by two authors [RM and HN], and a third was consulted where discrepancies arose [QV/IO].

### Data extraction

A data extraction sheet for included studies captured: study citation (author, year of publication); location; study setting; study design; number of participants; characteristics of participants; entrainment method; frequency of entrainment; time of exposure; comparison/control group; outcome measures; timing of measurement; primary outcome; secondary outcome and final conclusions.

### Meta-analysis

Inclusion criteria. We used four criteria for including a study in the meta-analysis: (1) was an experimental study; (2) used frequency stimulation in a sensory modality; (3) had control treatment; and (4) provided sufficient information to extract effect sizes. All nine studies met these criteria. We used a plot digitizer (https://automeris.io/WebPlotDigitizer/index.html) to extract the information needed from descriptive data plots for three studies.^20,29,30^

Statistical analysis. We used a random-effects model with restricted maximum likelihood estimation (REML) to measure the effect of entrainment on pain perception. We compared the standardised mean difference between the experimental (entrainment) and control treatment, using Hedges’ *g* as the effect size measure. We report *I*^2^ and *τ*^2^ as measures of between-study heterogeneity. For eight of the nine studies, we were able to explore the effect of entrainment on pain perception in different conditions, giving rise to 26 effect sizes. Additionally and for exploratory purposes, we used subgroup analysis for different outcomes measures including (1) fentanyl requirements during surgery; (2) perceived pain intensity based on VAS or numeric rating scale; and (3) Event-Related Potentials (ERPs) time-locked to laser-evoked pain. Finally, we present funnel plots with standard error of effect sizes^31^ and the Egger regression test^32^ to rule out possible publication bias. The meta-analysis was conducted using the meta (version 5.0–1^33^) and metasens (version 1.0–1^33^) packages for R Studio (version 1.4.1106). The data and script for all analyses are available at the Open Science Framework (osf.io/s64vj) or upon request to the authors.

### Critical appraisal

Included studies were appraised using the Critical Appraisal Skills Programme checklist for randomised controlled trials, which assesses the methodological quality of the study and the strength of results based on randomisation, blinding, sample size, baseline characteristics, reporting of results and value of the results. There were no ethical issues identified in conducting this review; therefore, no applications for approval were sought.

## Results

**Figure 1.**
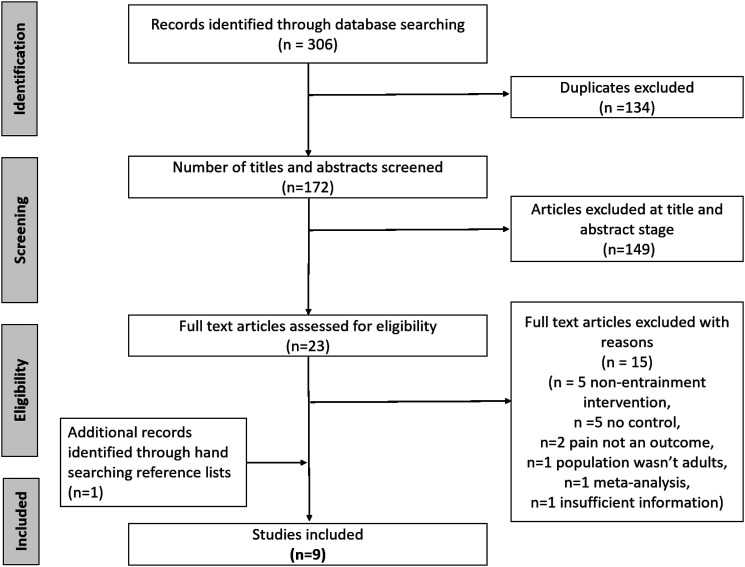
The PRISMA flow diagram of the search strategy and study selection process.

From 306 returned studies, nine studies met the inclusion criteria as illustrated in the PRISMA flow diagram of Figure 1 (see also Table 1). Three of these studies^34–36^ overlapped with a previous meta-analysis.^25^Tables 2 and 3 provide further summary details about the nine studies.

**Table 2.** A summary of nine studies that met the inclusion criteria and were included for analysis.

Reference	Study design	Study setting location	Number of participants	Condition
Kliempt et al., 1999	Randomised controlled trial	Hospital, UK	76	Surgery requiring general anaesthesia
Lewis et al., 2004	Randomised controlled trial	Hospital, USA	60	Bariatric or lumbar surgery
Dabu-bondac et al., 2010	Randomised controlled trial	Hospital, USA	60	Surgery requiring general anaesthesia
Zampi., 2015	Randomised repeated measures crossover controlled trial	Laboratory, USA	32	Chronic pain
Ecsy et al., 2017	Randomised repeated measures crossover controlled trial	Laboratory, UK	64	Healthy
Ecsy et al., 2018	Randomised repeated measures controlled trial	Laboratory, UK	32	Healthy
Arendsen et al., 2020	Randomised repeated measures crossover controlled trial	Laboratory, UK	20	Chronic pain
Gkolias et al., 2020	Double-blind randomised repeated measures crossover controlled trial	Pain unit and patient homes, Greece	21	Chronic pain
Olcucu et al., 2021	Randomised controlled trial	Hospital, Turkey	352	Cystoscopy and ureteral stent removal surgery patients

**Table 3.** A summary of study characteristics for the nine included studies.

Author, year	Intervention			Control	Measurement		Outcomes		Final conclusions
	Entrainment method	Neural oscillation frequency	Timing		Tools	Timing	Primary	Secondary	
Kliempt et al., 1999	Binaural beats	Unknown	During general anaesthesia	Classical music, blank tape	Fentanyl requirement (μg)	During general anaesthesia	Intervention group required mean 96 μg less fentanyl than classical music control (95 CI 64.6 μg–127.4 μg, *p* < 0.001) and mean 98 μg less fentanyl than blank tape control (95% CI 67.4 μg–128.6 μg, *p* < 0.001)	*n*/a	Patients required less analgesia while listening to binaural beats
Lewis et al., 2004	Binaural beats	Unknown	During general anaesthesia	Blank tape	Fentanyl requirement (μg/kg/min)	During general anaesthesia	Intervention group for bariatric surgery required less fentanyl than control (0.015 ± 0.01 vs 0.024 ± 0.01, *p* = 0.009), no difference in fentanyl requirements between intervention and control in lumbar group	n/a	Binaural beats may be a promising, novel, intra-operative, supplement to analgesia
Dabu-bondac et al., 2010	Binaural beats	Unknown	30 min pre-operative and during general anaesthesia	Music of choice, blank tape	Analgesic consumed (IV morphine equivalent, mg) VAS	Intra-operative, 24 h post-operative	Intervention group required less fentanyl compared to controls (14.6 ± 6.9 vs 20.9 ± 9.1 vs 20.3 ± 9.4, *p* = 0.046), no significant difference in post-operative analgesic use between intervention and controls	Intervention group had lower post-operative VAS scores at 1 h (2.6 ± 1.6 vs 4.2 ± 2.1 vs 3.9 ± 1.7, *p* = 0.02) and 24 h (3.5 ± 1.5 vs 5.3 ± 1.8 vs 5.0 ± 2.0, *p* = 0.005)	Binaural beats decreased consumption of intra-operative analgesia and decreased post-operative pain scores
Zampi, 2015	Binaural beats	6 Hz	20 min each day for 14 days	300 Hz tone	West haven-yale multidimensional pain inventory (MPI)	Following 14 days of intervention	Perceived pain intensity decreased following intervention (M = 4.60 to M = 2.74, *p* < 0.001)	n/a	Theta binaural beats were effective in reducing pain severity in chronic pain patients
Ecsy et al., 2017	LED goggles and binaural beats	8 Hz, 10 Hz, 12 Hz	10 min	1 Hz, white noise	Numeric rating scale	Immediately following intervention	Binaural beats lowered NRS scores compared to control, adjusted differences 8 Hz–0.52, (*p* < 0.001), 10 Hz–0.58 (*p* < 0.001), 12 Hz–0.51 (*p* < 0.001) LED lowered NRS compared to control, adjusted differences 8 Hz–0.60 (*p* < 0.01), 10 Hz–1.12 (*p* < 0.001), 12 Hz–0.35 (*p* < 0.01)	n/a	Visual and auditory entrainment can influence the perception of pain
Ecsy et al., 2018	LED goggles	8 Hz, 10 Hz, 12 Hz	10 min	1 Hz	Cerebral alpha power and laser-evoked potential (LEP)	Immediately following intervention	Alpha power increased following 8 Hz (*p* < 0.05) and 10 Hz (*p* < 0.05) visual entrainment, no significant effect of 12 Hz stimulation observed Significant change in P2 amplitude following 10 Hz entrainment (*p* < 0.001) but not following 8 Hz and 12 Hz entrainment Average reduction in P2 peak following 10 Hz entrainment compared to control was −2.33 μV (95% CI −4.29 to −0.37)	n/a	Induction of increased alpha power suppresses the cortical processing of acute pain
Arensden et al., 2020	LED goggles	10 Hz	4 min	1 Hz, 7 Hz	Global alpha power (GAP) numeric rating scale	Immediately following intervention	Alpha power significantly higher during 10 Hz stimulation compared to 1 Hz control during standing position (*p* < 0.001, no significant increase during sitting condition Alpha power significantly higher during 10 Hz stimulation compared to 7 Hz control in both sitting (*p* = 0.002) and standing (*p* < 0.001) conditions Global alpha power significantly higher during 10 Hz stimulation in standing condition compared to sitting (*p* < 0.001)	No significant change in pain intensity rating between intervention and control	4 min period of stimulation can increase alpha power, not sufficient to decrease chronic pain
Gkolias et al., 2020	Binaural beats	5 Hz	30 min (first phase) on demand for 7 days	400 Hz	Numeric rating scale defined daily doses (DDDs) STAI	Immediately following intervention (first phase) at the end (second phase)	NRS reduced following binaural beats (5.6 ± 2.3 to 3.4 ± 2.6, *p* < 0.001) compared to control (5.2 ± 2.1 to 4.8 ± 2.3, *p* = 0.79) in the first phase NRS reduced following binaural beats (5.6 ± 2.3 to 3.9 ± 2.5, *p* < 0.001) compared to control (5.2 ± 2.1 to 5.5 ± 2.6, *p* = 0.83) in the second phase Mean daily NRS was significantly reduced in BB intervention (6.9 ± 1.8 to 5.9 ± 1.8, *p* < 0.05) compared to control (6.4 ± 2.0 to 6.3 ± 1.7, *p* = 0.087)	DDDs reduced during BB intervention (4.8 ± 4.3 to 3.9 ± 3.7, *p* < 0.05) compared to control (4.7 ± 4.8 to 4.6 ± 4.1, *p* = 0.78) Stress significantly reduced in both interventions after 30 min, only in BB intervention after 7 days (46.8 ± 13.3 to 39.8 ± 11.5, *p* < 0.001)	Binaural beats significantly reduced pain intensity and analgesic consumption in chronic pain patients
Olcucu et al., 2021	Binaural beats	10 Hz	10 min before surgical intervention	Classical music, no audio	Visual analogue scale STAI	Following procedure	Significantly lower VAS scores for BB intervention in DC group compared to classical music control (2.67 ± 2.16 vs 3.72 ± 2.23, *p* < 0.0.11) and no audio control (2.67 ± 2.16 vs 4.69 ± 2.4, *p* < 0.001) Significantly lower VAS scores for BB intervention in USR group compared to classical music control (3.17 ± 1.93 vs 4.36 ± 2.23, *p* = 0.01) and no audio (3.17 ± 1.93 vs 5.67 ± 2.41, *p* < 0.001)	Significant decreases in STAI-T scores in BB intervention compared to no audio for DC group (36.04 ± 7.06 vs 40.69 ± 8.16,*p* = 0.001) and USR group (36.19 ± 7.06 vs 40.90 ± 8.00, *p* = 0.007) No significant difference between BB and classical music groups for both DC and USR groups. Significant decreases in STAI-D scores in BB intervention when compared to no audio for DC group (36.04 ± 7.06 vs 40.69±8.16, *p* = 0.001) and USR group (5.78±2.74 vs 1.13 ± 0.79, *p* < 0.001), no significant difference between BB and classical music groups for both DC and USR groups	Binaural beats are a simple and effective method to reduce anxiety and pain scores associated with diagnostic cystoscopy and ureteral stent removal procedures

### Study characteristics

Six studies investigated binaural beats as the entrainment method.^28,34–38^ Two studies investigated LED goggles/visual stimulation^20,30^ and one study investigated both binaural beats and LED goggles.^39^

Four studies investigated alpha frequencies.^20,30,38,39^ Of these, two investigated 8-, 10- and 12 Hz stimulation frequencies^20,39^ and two investigated 10 Hz stimulation frequency.^30,38^ Two studies investigated theta frequency entrainment.^28,37^ Of these, one investigated 5 Hz frequency stimulation^37^ and the other investigated 6 Hz stimulation.^28^ One study investigated alpha (10 Hz) and theta (7 Hz) visual stimulation, but the authors considered theta stimulation as an additional control along with a 1 Hz frequency stimulation (see Table 3).^30^ The three remaining studies investigated Hemi-Sync® (i.e. hemispheric synchronisation^40^) as the stimulation method.^34–36^ This method present different sounds including binaural beats, but the frequencies contained in the hemi-sync sounds were not reported.

Four studies were undertaken in the United Kingdom,^20,30,34,39^ three in the United States, ^28,35,36^ one in Greece^37^ and one in Turkey.^38^ Four of the nine studies investigated patients undergoing surgery,^34–36,38^ three involved volunteers with chronic pain,^28,30,37^ and two involved healthy volunteers.^20,39^

Three studies investigated analgesia requirements during general anaesthesia as their primary outcome.^34–36^ Fentanyl requirements during surgery in micrograms^34^ and micrograms/kilogram/minute^35^ and intravenous morphine equivalent in milligram^36^ were used as the outcome measures. Fentanyl was administered intravenously if heart rate and/or blood pressure increased more than a fixed percentage of pre-operative baseline (e.g. 15%–20%) until these measurements returned to baseline. The dosage was used as a proxy of nociceptive control.

Four studies investigated pain intensity following entrainment.^28,37–39^ Two studies investigated pain intensity following entrainment as a secondary outcome.^30,36^ Two studies measured pain using a visual analogue scale.^36,38^ Three studies measured pain using a numeric rating scale.^30,37,39^ One study measured pain using the West Haven-Yale Multidimensional Pain Inventory.^28^ Two studies investigated alpha power following entrainment,^20,30^ one of which also investigated changes to the amplitude of the N2 and P2 components of laser-evoked potentials in EEG data.^20^ The amplitude of these components are correlated with subjective pain intensity.

### Outcome of meta-analysis

**Figure 2.**
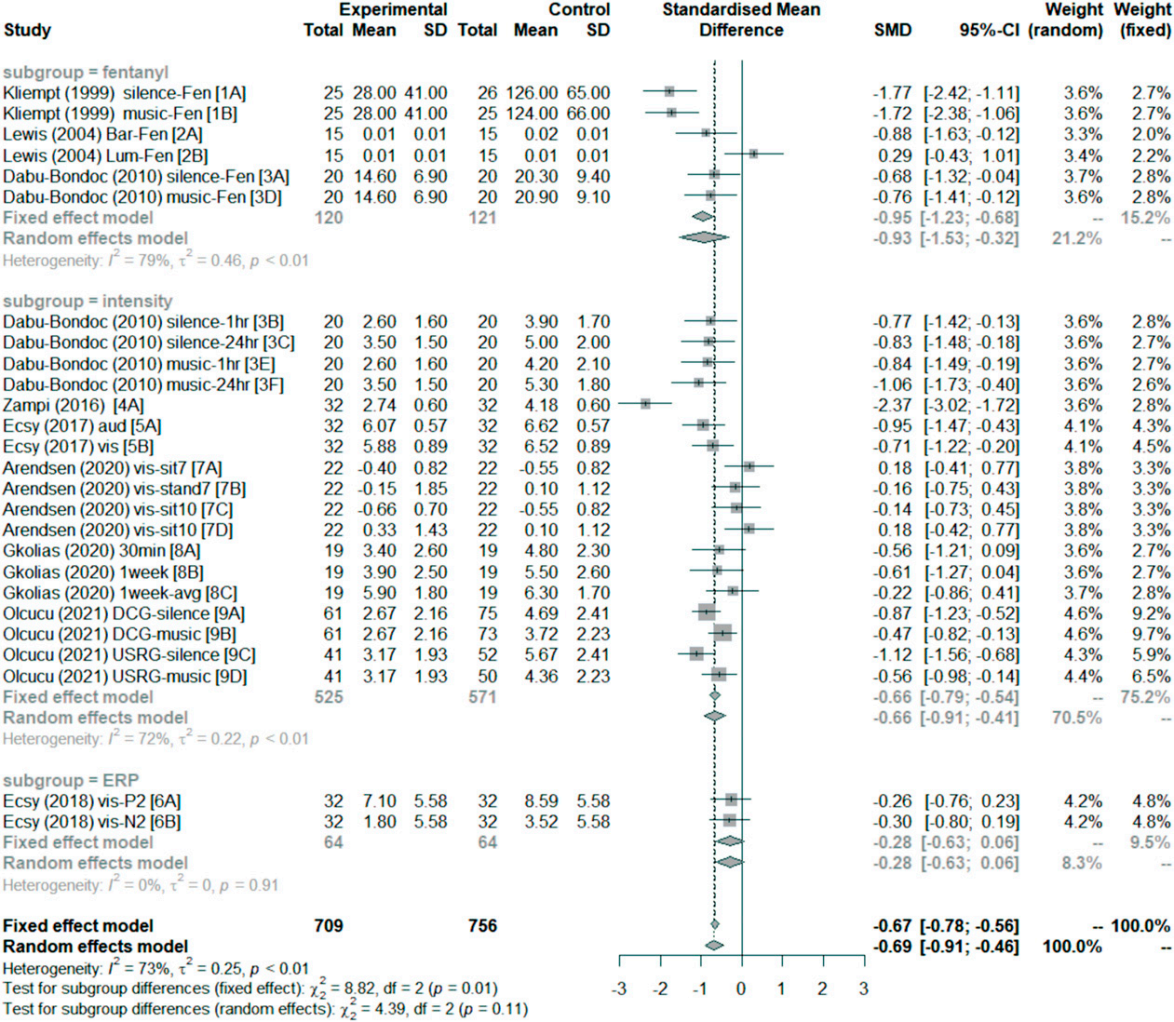
Forest plot of effect size estimates (Hedges’ *g*) divided by subgroups. Negative effect sizes favour sensory entrainment. Horizontal lines depict 95% confidence interval (95% CI), size of squares represents the weight of individual data sets based on sample size, and diamonds represent mean effect sizes. Both fixed-effect and random-effects models are presented for completeness, but this meta-analysis focused on the random-effects model. The fentanyl subgroup measured fentanyl requirements for surgery related to pain perception. The intensity subgroup measured pain intensity perception using visual analogue scales or numeric ratings. The ERP subgroup measured the amplitude of ERP components related to pain perception. The vertical dashed line represent overall mean effect size under the fixed-effect model.

Figure 2 summarises the results of the meta-analysis in a forest plot. Overall, sensory entrainment decreased pain perception (effect size: mean = −0.69, 95% confidence interval = [−0.91–0.46]). As indicated by the subgroup analysis, this decrease was found for pain intensity rating;^28,30,36–39^ and fentanyl requirement during surgery,^34–37^ but not ERP response to laser-evoked pain.^30^ A *χ*^2^ test showed no significant subgroup effect under the random-effects model (*χ*^2^ = 4.39, *p* = 0.11; but there was one under the fixed-effect model, *χ*^2^ = 8.82, *p* = 0.01). There are indications that some studies or conditions may not show a change in pain perception with sensory entrainment compared to a control treatment. Arendsen et al.^30^ did not find a decrease in pain intensity rating while participants were standing after receiving visual alpha entrainment or sitting after receiving visual theta entrainment relative to 1 Hz visual stimulation. Lewis et al.^35^ did not observe any differences between listening to binaural beats and listening to a blank tape (silence) for patients undergoing lumbar disk surgery.

**Figure 3.**
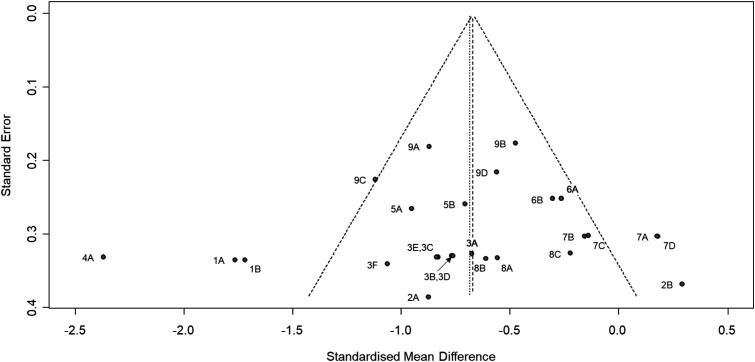
Funnel plot of effect size estimates (Hedges’ *g*). The effect size estimates are shown with 95% confidence interval relative to standard error for individual data sets. The point labels correspond to the study labels in Figure 2. The left-most vertical line represent the standardised mean difference under the random-effects model, and the other vertical line (under the triangle) represent the standardised mean difference under the fixed-effects model.

Although there are entrainment effects, the heterogeneity was generally large. This is the case across all studies (*I*^2^ = 73% and *τ*^2^ = 0.25, *p* < 0.01), and for the fentanyl (*I*^2^ = 79% and *τ*^2^ = 0.46, *p* < 0.01) and intensity (*I*^2^ = 72% and *τ*^2^ = 0.22, *p* < 0.01) subgroups. There were too few effect sizes for the ERP subgroup (see Figure 2). Finally, the funnel plot in Figure 3 indicates that there was no evidence of publication bias (Egger test: *t*(24) = −0.39, *p* = 0.70).

### Critical appraisal

The included studies adopted parallel group randomised controlled trials^34–38^ and repeated measures crossover design.^28–30,39^ The studies were generally of high quality and were therefore included in the meta-analysis. The most common limitation was the lack of detail around blinding of participants and researchers.^28–30,34,38,39^

## Discussion

Our systematic review and meta-analysis suggest that sensory entrainment is associated with reduced overall perceptual (intensity) and proxy (fentanyl requirement) outcome measures in pain studies. Sensory entrainment, however, was not associated with consistent changes to ERP-response proxy outcome measures in these studies. Both visual and auditory stimulation methods decreased pain intensity ratings; additionally, binaural beats also reduced both the fentanyl requirements needed during surgery and post-operative pain ratings. Our review further suggests that different neural oscillation frequencies may have different efficacy for acute compared to chronic pain. Specifically, whilst neural entrainment of alpha frequencies was effective in reducing pain intensity from experimentally induced acute pain, there was no consistent evidence supporting its use in chronic pain. A recent study^41^ published after our search period extended Arendsen et al.’s^30^ study by testing eight additional volunteers, thereby increasing statistical power. This study provided evidence that visual alpha entrainment may reduce pain intensity ratings for those with chronic pain. In contrast, neural entrainment of theta frequencies was effective in reducing pain intensity from chronic pain, but there was no evidence for reducing pain intensity from acute pain. Moreover, several studies included in our review demonstrated that visual and auditory entrainment modulated power at the stimulation frequency.^20,30,39^ Although there is promising evidence for the efficacy of sensory entrainment in reducing acute and chronic pain, our review highlights that the current evidence is not homogenous and varies in strength between studies.

### Effectiveness of alpha (8–13 Hz) neural entrainment in acute pain

A key finding of our review suggests that neural entrainment of alpha frequencies by visual and auditory stimulation methods was effective in reducing acute pain. In particular, visual or auditory entrainment at 8, 10 or 12 Hz resulted in lower pain intensity ratings for acute pain induced by laser stimulation or during surgery relative to a control sensory stimulation.^38,39^ These results complement EEG studies which found a reduction of alpha power during painful stimulation.^6,9,42,43^ That is in line with our hypothesis, we expect that increasing power in alpha frequencies by sensory entrainment would lead to a corresponding reduction in pain intensity.

The subgroup analysis of visual alpha entrainment on the ERP response revealed no consistent evidence for an entrainment effect when pooled over the P1 and P2 components.^20^ Interestingly, Ecsy et al.^20^ found that the 10 Hz visual entrainment was shown to result in a reduction of the P2 peak amplitude, which may reflect cortical responses to nociception.^44^ This was observed in the insular cortex where the magnitude of perceived pain from a noxious stimulus is believed to be encoded,^45^ as well as in the posterior cingulate cortex and the precuneus. Reductions in P2 peak amplitudes in these brain structures indicate that they may be involved in alpha entrainment induced pain suppression, possibly via a mechanism in which increased alpha power leads to a change in attention towards the painful stimulation. Indeed, the reduction in P2 amplitude is consistent with evidence of alpha oscillations involvement in top-down inhibition of activity in sensory networks^9,46^ and the correlation between an increase in alpha power and deactivation in cortical areas.^19,47^ We note that ERP responses may reflect attentional capture by nociceptive stimulation (i.e. stimulus saliency) rather than perceived pain intensity.^48^ Thus, more research is needed to determine whether ERP response can be used as an outcome measure for pain perception, for example, by investigating different components of the ERP response.

It can be postulated that alpha entrainment leads to the inhibition or deactivation of cortical areas involved in pain processing and a subsequent reduction in pain perception. Alpha oscillations are also involved in an attentional suppression mechanism.^49^ Attention is known to influence pain perception and pain is perceived as more intense when focused on.^50^ The increase in alpha power following entrainment could suppress attention towards the painful stimulus which is then perceived as less intense.

In contrast to acute pain, the findings by Arensden et al.^30^ suggest that 10 Hz visual entrainment may not be effective in reducing pain intensity in patients with chronic musculoskeletal pain. This study may be underpowered (see^41^). It may also be possible that chronic-pain-induced neural changes can influence the efficacy of alpha entrainment in reducing pain.^45,51^ Moreover, changes in the oscillatory activity observed in chronic-pain patients such as increases in theta oscillations^12,52^ may also contribute to the lack of efficacy observed by Arensden et al.^30^

### Effectiveness of theta (4–7 Hz) neural entrainment in chronic pain

There was no consistent evidence that visual entrainment reduced pain intensity for chronic pain.^30^ By comparison, auditory theta entrainment was shown to be effective in reducing chronic pain.^28,37^ The results from these studies are surprising as chronic pain has been characterised by an increase in theta power due to altered thalamo-cortical activity.^12,53^ Moreover, in participants with no chronic-pain conditions, painful laser stimulation resulted in significantly higher theta power than intensity-matched innocuous tactile or electrical stimulation.^11,54^ As entrainment increases theta power, it would not be expected to reduce pain intensity ratings for those with chronic pain. Gkolias et al.^37^ speculated that a more distributed network of brain structures entrained to theta frequency might enhance overall neural inhibition that is reduced during thalamo-cortical dysrhythmia resulting in pain reduction. The reduction in pain intensity following theta entrainment despite the observations of increased theta activity in acute and chronic pain demonstrates the need for a better understanding of the neurological processes associated with chronic pain.

Importantly from a clinical perspective, a significant reduction in analgesic medication use was observed when theta entrainment was used on demand over several days.^28,37^ This is an important finding given the lack of evidence for the efficacy of long-term use of medications as well as potential adverse effects associated with them.^1,2,55^ Three studies used the Hemi-Sync® method^40^ to present different sounds including binaural beats, but the specific sounds and frequencies were not reported.^34–36^ That said, listening to hemi-sync sounds reduced post-operative pain ratings and time until discharge following administration 30 min before surgery and during general anaesthesia.^36^ Listening to hemi-sync sounds also decreased the amount of fentanyl patients needed during surgery.^34–36^ These studies were also analysed by Peng and Tang^25^ who focused on fentanyl requirements and did not consider pain intensity, nor did the researchers consider other sensory modalities. The reduction in analgesic requirements would improve patient safety and so this, along with the pain reductions, demonstrate the potential clinical usefulness of sensory theta entrainment.

### Limitations of the evidence

Despite the potential for clinical use of sensory neural entrainment identified in this review, there are several limitations which may influence the conclusions drawn from our study. First, the number of studies included in this study is low and the evidence base is limited. A greater number of studies will be necessary to provide a more robust evaluation of the practical application of sensory entrainment specifically on pain perception.

Secondly, all of the studies with the exception of one, had a modest sample size (<80). Further studies in this area should include a larger sample size to ensure studies are appropriately powered and provide reliable data. A larger sample size may also allow assessment of how psycho-social factors affect the efficacy of sensory entrainment methods in clinical settings in which patients have a large variability of such factors.^56^ Given this variability in patient cohorts, small sample sizes may be more problematic for clinical studies. For example, Arendsen et al.’s^30^ study may have been underpowered to detect a consistent effect size of sensory entrainment for patients with chronic musculoskeletal pain (see also^41^).

Lastly, no studies directly compared the effect of sensory entrainment on healthy individuals with individuals suffering from chronic pain. Evidence suggests that chronic pain has an impact on brain function and structure.^45,51^ While this review included studies investigating chronic-pain patients and healthy volunteers, there was no study identified which included both groups.

## Conclusions and future directions

In conclusion, sensory entrainment is a promising neuro-modulatory method for reducing pain perception in adults. Indeed, the acceptability and usability of auditory and visual entrainment using a smartphone app was supported by chronic-pain patients who welcomed it as an alternative to medication,^15^ but more experimental work is needed to ensure safe and valid use of sensory entrainment.^57^ The included studies support the efficacy of auditory and visual alpha entrainment in reducing acute pain, and auditory theta entrainment in reducing chronic pain. We also identified that auditory entrainment can reduce intra-operative analgesic requirements and post-operative pain. These conclusions should be considered in light of the small number of studies, and small number of participants and patients. Furthermore, given the heterogeneity of studies, there is insufficient evidence to determine the relative strengths of different sensory modalities and whether the efficacy of sensory entrainment differs between acute and chronic pain.

Further research is required regarding the timing, duration and frequency of the intervention to determine the best application for maximum efficacy. More studies with larger sample sizes and double-blinded randomisation would also be necessary before implementation into clinical care of pain. Overall, given the lack of efficacy of available pharmacological treatments for pain, our systematic review and meta-analysis suggest that sensory entrainment could provide a valuable non-pharmacological alternative for pain management.
